# Proton Gradient-Dependent Transport of *p*-Glucocoumaryl Alcohol in Differentiating Xylem of Woody Plants

**DOI:** 10.1038/s41598-019-45394-7

**Published:** 2019-06-20

**Authors:** Taku Tsuyama, Yasuyuki Matsushita, Kazuhiko Fukushima, Keiji Takabe, Kazufumi Yazaki, Ichiro Kamei

**Affiliations:** 1 Faculty of Agriculture, University of Miyazaki, Miyazaki, 889-2192 Japan; 2 Graduate School of Bioagricultural Sciences, Nagoya University, Nagoya, 464-8601 Japan; 3 Graduate School of Agriculture, Kyoto University, Kyoto, 606-8502 Japan; 4 Research Institute for Sustainable Humanosphere, Kyoto University, Uji, 611-0011 Japan

**Keywords:** Secondary metabolism, Plant physiology, Membrane proteins, Secretion, Cell wall

## Abstract

Lignin is a cell wall component of vascular plants crucial for survival in terrestrial environments. While *p*-hydroxyphenyl lignin is minor, it is considered to be localised in the outermost part of the cell wall providing strong adhesion between cells, which determines cell shape. Transport of the lignin precursor from the cytosol to the cell wall is critical to regulate temporal and spatial lignin deposition; however, little information on the transport step is available. Here, we report transport activity of *p*-glucocoumaryl alcohol, a precursor of *p*-hydroxyphenyl lignin, in a broad-leaved tree (hybrid poplar, *Populus sieboldii* × *P*. *grandidentata*) and a coniferous tree (Japanese cypress, *Chamaecyparis obtusa*). Membrane vesicles of both trees were prepared from differentiating xylem with vigorous lignification and used for transport assays. Several inhibition assays indicated that not ABC transporters but the proton gradient and V-ATPase are involved in *p*-glucocoumaryl alcohol transport depending on ATP. These results support the hypothesis that *p*-glucocoumaryl alcohol is loaded into the secretory vesicles and delivered to the cell wall by exocytosis. Furthermore, this transport mechanism was common in both poplar and Japanese cypress, strongly suggesting that *p*-glucocoumaryl alcohol transport in the differentiating xylem is conserved within woody plants.

## Introduction

Lignin is a cell wall component of vascular plants vital for survival in terrestrial environments through its contribution to efficient water flow, resistance to biotic attack, UV stress protection, and the compressive strength that controls plant architecture. Lignin is composed of three types of monomers: *p*-hydroxyphenyl (H), guaiacyl (G), and syringyl (S) units. Lignification occurs mainly in the differentiating xylem during secondary cell wall formation. Deposition of lignin is initiated at the cell corner and in the compound middle lamella, i.e., middle lamella and primary cell wall^1,2^.

*p*-Hydroxyphenyl lignin (H lignin) is a minor component especially in gymnosperms and dicotyledons and its physiological role is unclear; however, the deposition manner of H lignin is different from G or S lignin, which suggests several roles specific to H lignin. It has been suggested that H lignin is deposited at the cell corner and in the compound middle lamella^3–5^ during a very early stage of lignification^6–8^, which is assumed to play a role in the rigidification of cell connections and the finalisation of shape of cells. Coniferous trees form reaction wood or compression wood in the lower trunk if inclined as a response to gravity; this wood is known to contain more H lignin than normal wood^9,10^, which indicates that H lignin contributes to generate compression strain. A considerable amount of *p*-glucocoumaryl alcohol, a glucoside of *p*-coumaryl alcohol which is a major monomer of H lignin, was detected in compression wood where H lignin content was higher than normal wood, suggesting that it is also involved in H lignin biosynthesis^11^.

Biosynthesis of lignin proceeds in three steps: (1) biosynthesis of lignin precursors within the cell, (2) transportation of the precursors to the cell wall, and (3) dehydrogenative polymerisation of the precursors in the cell wall. Although steps (1) and (3) have been intensively studied^12–14^, much less is known about how the lignin precursors are transported to the cell wall.

Biochemical experiments using microsomal fractions from rosette leaves of *Arabidopsis thaliana* indicated the possible involvement of an ATP-binding cassette (ABC)-like transporter in the transport of lignin precursors^15^. In *A*. *thaliana* leaves, however, only a limited number of cells undergo lignification, whereas the differentiating xylem of woody plants shows vigorous lignification and thus is suitable experimental material for investigation of lignin-precursor transport. Studies of differentiating xylem of angiosperms and gymnosperms demonstrated that transport of coniferin, a glucoside of coniferyl alcohol as one of the G lignin precursors, is dependent on ATP, and mediated by a proton gradient formed by vacuolar-type H^+^-ATPase (V-ATPase), which is a common mechanism in hybrid poplar and Japanese cypress^16^. Considering the localisation of coniferin *β*-glucosidase in the cell wall in lodgepole pine^17^ and poplar^18^, it was hypothesized that coniferin is transported into the secretory vesicles, by which coniferin is supplied to the developing cell wall by exocytosis^16,18^. Transport of H lignin precursors may regulate temporally and spatially specific deposition of H lignin, resulting in the finalisation of shape and size of cells in the differentiating xylem or generating compression strain. However, no information on the biochemical transport activity of the H lignin monomer in plants is available.

In the present study, H lignin formation in differentiating xylem of poplar was revealed by thioacidolysis. Furthermore, we demonstrated that *p*-glucocoumaryl alcohol is transported into the tonoplast and endomembrane vesicles of differentiating xylem in poplar. The transport activity is proton gradient-dependent across the tonoplast and endomembrane compartments, which is consistent with that of coniferin transport previously reported. Our results suggest that a proton antiporter in the tonoplast and endomembrane is involved in transporting *p*-glucocoumaryl alcohol and coniferin in the differentiating xylem where lignification proceeds vigorously.

## Results

**Figure 1:**
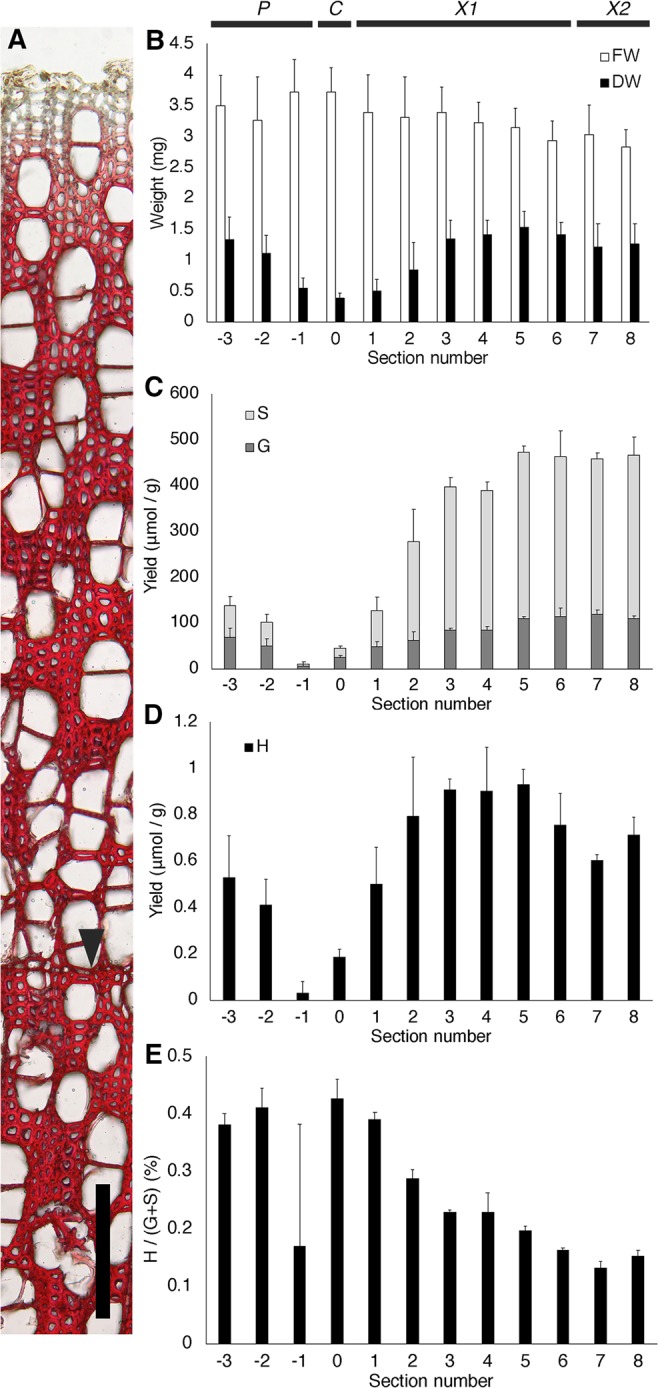
(**A**) Transverse section of poplar differentiating xylem stained using the Mäule reaction. Top, debarking position; arrow head, boundary of annual ring; bar = 200 µm. (**B**) Fresh and dry weight of tangential sections prepared from blocks of poplar wood. Region P, phloem; C, cambium and cell expansion zone; X1, xylem formed in the present year; X2, xylem formed in the previous year. (**C**,**D**) Syringyl and guaiacyl monomer (**C**) and *p*-hydroxyphenyl monomer (**D**) yield by thioacidolysis. (**E**) Distribution of H/(G + S) ratio in the radial direction of poplar determined by thioacidolysis. Data are the mean ± SD (B, *n* = 6; C–E, *n* = 3).

Lignification mostly occurs in the differentiating xylem of trees. Staining of transverse sections using the Mäule reaction demonstrated vigorous lignin deposition in poplar differentiating xylem (Fig. 1A). To detect lignin and H lignin deposition, approximately 200-µm-thick serial tangential-section samples (sections −3 to 8, Fig. 1) were prepared from poplar wood blocks containing phloem, differentiating xylem and mature xylem. From the bark side the dry weight of the sections gradually decreased towards section 0 and then gradually increased towards section 3, where it remained constant (Fig. 1B). The annual ring was located within the sections 1,000–1,200 µm from the debarking position (Fig. 1A); the boundary of the annual ring was indicated to be around section 6. The above results indicated that section 0 contained the cells of the cambium and cell expansion zone, and that cell wall formation in the xylem ceased around section 3 (Fig. 1B).

Thioacidolysis analysis showed that G and S lignin were deposited in accordance with xylem differentiation (Fig. 1C). *p*-Hydroxyphenyl lignin deposition was detected in poplar samples, although the yield was very low (Fig. 1D). The ratio of H units to G and S units peaked at an early stage of xylem formation and decreased as xylem formation proceeded (Fig. 1E).

**Figure 2:**
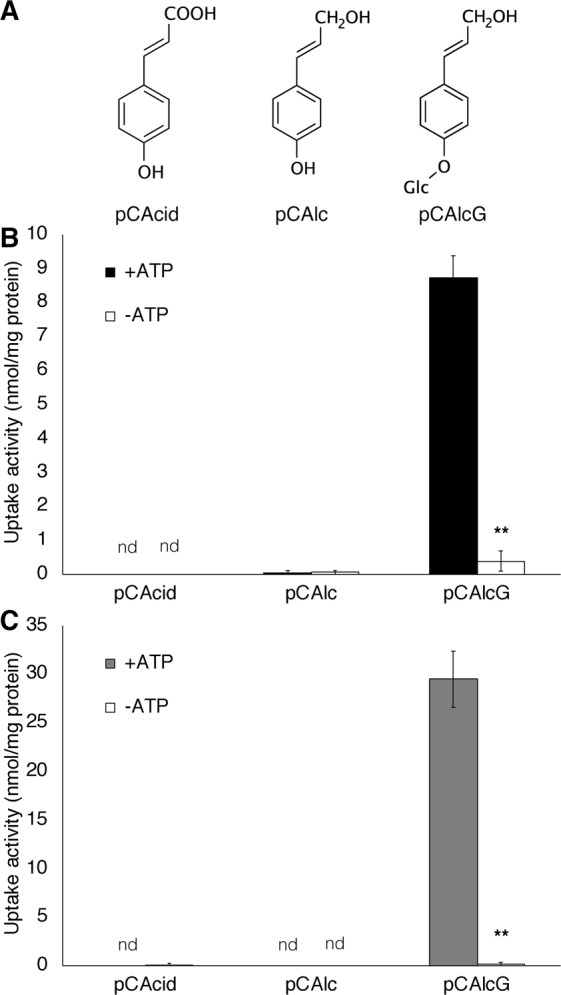
Uptake of H lignin precursors into membrane vesicles. (**A**) Chemical structure of H lignin precursors tested in the present study. (**B**) and (**C**) show uptake activities of lignin precursors in membrane vesicles isolated from differentiating xylem of hybrid poplar and Japanese cypress, respectively. Membrane vesicles were incubated with 50 *μ*M of each compound in the presence or absence of 5 mM Mg/ATP for 20 min. pCAcid, *p*-coumaric acid; pCAlc, *p*-coumaryl alcohol; pCAlcG, *p*-glucocoumaryl alcohol. Data are the mean ± SD of three replicates. **P < 0.01 compared with/without ATP using Student’s *t*-test.

To elucidate the transport mechanisms of H lignin precursors we sampled differentiating xylem tissues from an approximately 40-year-old hybrid poplar^16^. The majority of cells of the sampled tissues were undergoing vigorous lignification. From these tissues, microsomal membrane vesicles were prepared for transport assays using three potential lignin precursors as substrates (Fig. 2A). Figure 2B shows the uptake of three kinds of H lignin precursors (pCAcid, *p*-coumaric acid; pCAlc, *p*-coumaryl alcohol; pCAlcG, *p*-glucocoumaryl alcohol) into the membrane vesicles obtained from the differentiating xylem of hybrid poplar. Distinct uptake of *p*-glucocoumaryl alcohol was observed in the presence of ATP. Almost no uptake was observed for the aglycones, i.e., *p*-coumaryl alcohol or *p*-coumaric acid. For comparison, we prepared microsomal vesicles from differentiating xylem of Japanese cypress, a coniferous tree, in which strong ATP-dependent transport activity of *p*-glucocoumaryl alcohol was observed (Fig. 2C). The membrane vesicles used in this study showed coniferin uptake activity as previously reported^16^, which was higher than the *p*-glucocoumaryl alcohol uptake activity in the membrane vesicles from differentiating xylem of both the hybrid poplar and Japanese cypress (Fig. S1).

**Figure 3:**
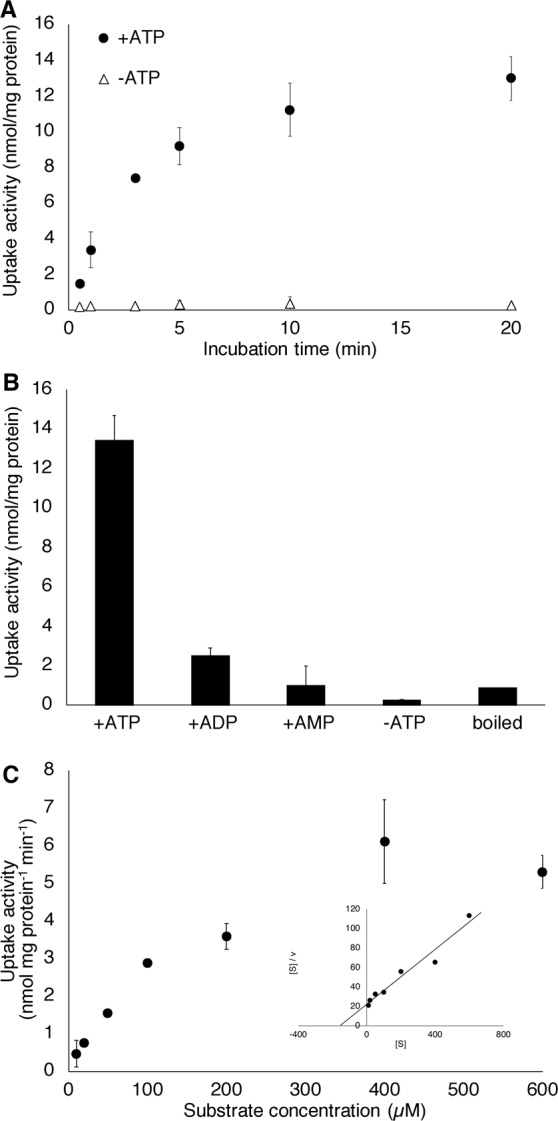
(**A**) Time course of *p*-glucocoumaryl alcohol uptake in hybrid poplar membrane vesicles. Membrane vesicles were incubated with 50 *μ*M *p*-glucocoumaryl alcohol in the presence (●) or absence (△) of 5 mM Mg/ATP. (**B**) Negative control for *p*-glucocoumaryl alcohol uptake. Membrane vesicles of hybrid poplar were incubated with 50 *μ*M *p*-glucocoumaryl alcohol for 20 min either with 5 mM Mg/ATP, with 5 mM Mg/ADP (+ADP), with 5 mM Mg/AMP (+AMP), or without Mg/ATP (−ATP). “Boiled” refers to a microsomal fraction heat-denatured for 10 min. (**C**) *p*-Glucocoumaryl alcohol uptake shows saturation kinetics. Membrane fractions of hybrid poplar were incubated for 5 min in the presence of 5 mM Mg/ATP and each concentration of *p*-glucocoumaryl alcohol. Inset shows Hanes–Woolf plots. Data are the mean ± SD of three replicates.

Rapid uptake of *p*-glucocoumaryl alcohol by membrane vesicles prepared from differentiating xylem of the hybrid poplar was dependent on ATP (Fig. 3A). Uptake of monolignol, *p*-coumaryl alcohol was not observed even with longer incubation (Fig. S2). Uptake activity of *p*-glucocoumaryl alcohol was not detected in the absence of ATP and was reduced if ADP or AMP was present (Fig. 3B), suggesting that ATP hydrolysis to ADP is the main energy resource for this activity. A heat-denatured microsomal fraction did not show transport activity. *p*-Glucocoumaryl alcohol transport activity by membrane vesicles of the hybrid poplar demonstrated Michaelis–Menten-type saturation kinetics (Fig. 3C). The apparent *K*_m_ value of *p*-glucocoumaryl alcohol transport in the hybrid poplar membrane vesicles was calculated to be 160–260 µM. The *K*_m_ value of *p*-glucocoumaryl alcohol is larger than that for coniferin transport (60–80 µM in poplar) or other previously reported transporters that function as secondary transporters (Table S1)^16,19–23^.

**Figure 4:**
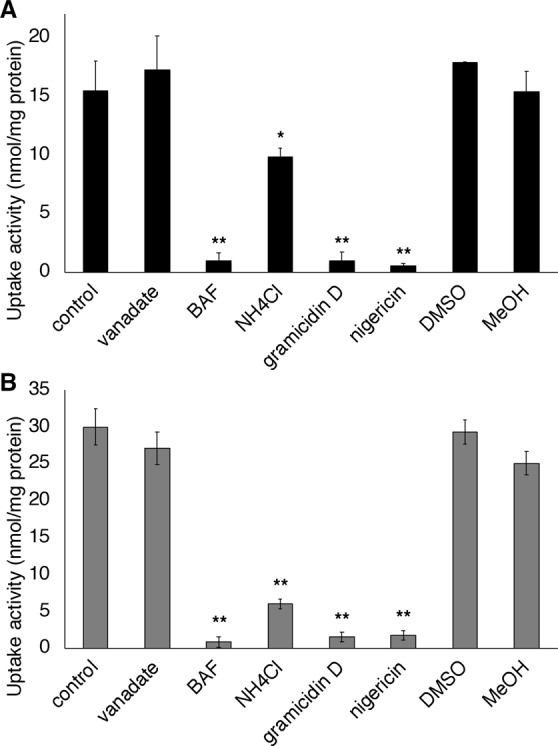
Effects of various inhibitors on *p*-glucocoumaryl alcohol transport in hybrid poplar (**A**) or Japanese cypress (**B**). Microsomal membranes were incubated for 20 min with 50 *μ*M *p*-glucocoumaryl alcohol and 5 mM Mg/ATP, to which vanadate (1 mM), bafilomycin A1 (BAF) (1 µM), NH_4_Cl (10 mM), gramicidin D (25 µM), or nigericin (20 µM) were added. Data are means ± SD of three replicates. *P < 0.05, **P < 0.01 compared with control (vanadate, NH_4_Cl), DMSO (BAF, gramicidin D), or MeOH (nigericin) by Student’s *t*-test.

For further characterisation of *p*-glucocoumaryl alcohol transport, a variety of inhibitors were used in transport assays. Bafilomycin A1 (BAF), a specific V-ATPase inhibitor, severely inhibited *p*-glucocoumaryl alcohol uptake by the membrane vesicles of the hybrid poplar (Fig. 4A). The proton gradient erasers, NH_4_Cl or the ionophores gramicidin D and nigericin, also inhibited *p*-glucocoumaryl alcohol transport activity. In contrast, vanadate, a common inhibitor of ABC transporters, showed no effect on *p*-glucocoumaryl alcohol transport. The same trends were observed in *p*-glucocoumaryl alcohol uptake in microsomal vesicles isolated from differentiating xylem of Japanese cypress (Fig. 4B).

**Figure 5:**
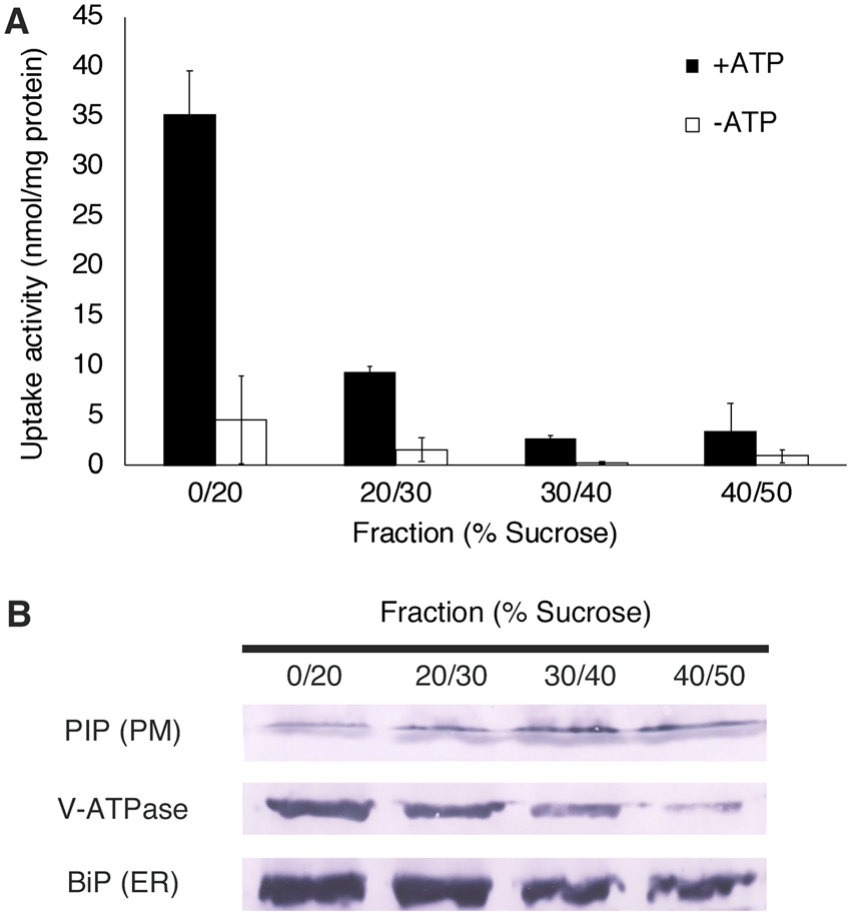
(**A**) *p*-Glucocoumaryl alcohol uptake activity of each membrane fraction collected from the interface between the indicated sucrose concentrations. Fractions were incubated with 50 *μ*M *p*-glucocoumaryl alcohol in the presence or absence of 5 mM Mg/ATP. Data are the mean ± SD of three replicates. (**B**) Plasma membrane intrinsic protein, V-ATPase and BiP were immunodetected to confirm the purity of plasma membrane vesicles, tonoplast and endomembrane vesicles, and ER membrane vesicles, respectively. Each marker protein was detected using a different membrane.

To elucidate the origin of the microsomes involved in *p*-glucocoumaryl alcohol transport, microsomal vesicles of the hybrid poplar were fractionated using a discontinuous sucrose-density gradient followed by ultracentrifugation. Uptake of *p*-glucocoumaryl alcohol dependent on ATP was clearly enriched in the 0/20% fraction (Fig. 5A). This result was similar to the V-ATPase distribution observed by immunodetection (Fig. 5B), whereas plasma membrane and endoplasmic reticulum (ER) membrane fractions determined from the immunodetection of plasma membrane intrinsic protein (PIP) and binding immunoglobulin protein (BiP), respectively, showed different distribution patterns from the uptake activity of *p*-glucocoumaryl alcohol. Estimation of central vacuole distribution by *α*-mannosidase activity^24^ also showed a different pattern from *p*-glucocoumaryl alcohol transport (Fig. S3).

To further evaluate the substrate specificity of the transporter, *cis*-inhibition experiments were performed, in which the influence of monolignol or glucosides on *p*-glucocoumaryl alcohol transport was examined. *p*-Glucocoumaryl alcohol transport was inhibited by high concentrations of coniferin and slightly influenced by the aglycone *p*-coumaryl alcohol, but not inhibited by syringin, a sinapyl alcohol glucoside, and sucrose (Table 1). Inhibition of *p*-glucocoumaryl alcohol transport by different concentrations of coniferin indicates that coniferin is a mixed-type inhibitor of *p*-glucocoumaryl alcohol transport (Fig. S4).

**Table 1:** *cis*-Inhibition of *p*-glucocoumaryl alcohol uptake.

Compounds	0 *μ*M (control)	50 *μ*M	250 *μ*M
pCAlc	1.00 ± 0.06	1.07 ± 0.13	0.72 ± 0.07^**^
coniferin	1.00 ± 0.05	0.90 ± 0.03^*^	0.41 ± 0.04^**^
syringin	1.00 ± 0.01	1.02 ± 0.03	0.95 ± 0.25
sucrose	1.00 ± 0.27	1.16 ± 0.07	1.05 ± 0.13

Membrane vesicles obtained from differentiating xylem of hybrid poplar were incubated with 50 *μ*M *p*-glucocoumaryl alcohol in the presence of 5 mM Mg/ATP and each compound with indicated concentration. pCAlc, *p*-coumaryl alcohol. Data are the mean ± SD of more than three replicates. *P < 0.05, **P < 0.01 compared with the control using Student’s *t*-test.

## Discussion

Lignification starts at cell corner middle lamellae and proceeds to the compound middle lamellae, which contain higher amounts of H lignin than that of other cell wall layers^3–8^. In the present study, thioacidolysis revealed H lignin formation in differentiating xylem of poplar. Poplar xylem contained very small amounts of H lignin, and the peak value of the H/(G + S) ratio was observed at an early stage of xylem formation (Fig. 1). These observations indicate that H lignin deposition mainly occurs at an early stage of xylem formation rather than a later stage, which is consistent with the previous study of poplar using radio-labelled *p*-glucocoumaryl alcohol^7^. *p*-Hydroxyphenyl lignin is a minor component of cell walls and its physiological role is unclear; however, it is assumed to perform critical roles in the compound middle lamella, such as aiding in cell rigidity. The H ratio pattern observed in differentiating xylem of poplar was different to that observed during bamboo shoot development^25^, which implies that the role of H lignin may differ among plant species.

To elucidate the innate mechanism of lignin-precursor transport for lignin biosynthesis, biochemical analysis is very important; however, no biochemical evidence for transport of H lignin precursors in plants is available. Thus, we conducted transport assays using microsomal vesicles from poplar differentiating xylem where vigorous lignification occurs. In this study, we observed ATP- and time-dependent transport of *p*-glucocoumaryl alcohol (Figs 2, 3). Though the values of the transport activity were varied depending on the isolated membrane vesicles, active transport of *p*-glucocoumaryl alcohol was obviously shown by every membrane vesicle. This transport activity was not limited only to poplar but also observed in membrane vesicles prepared from Japanese cypress (Fig. 2C). Thus, *p*-glucocoumaryl alcohol transport dependent on ATP may be common in differentiating xylem of both gymnosperms and angiosperms.

In contrast, uptake of monolignol aglycones by the membrane vesicles was not observed, regardless of the presence of ATP (Figs 2, S2), indicating that *p*-coumaryl alcohol is not transported or diffused across membranes of the differentiating xylem. Although a study on model membranes demonstrated partitioning of lignin precursor models into the membrane^26^, the present results indicate that transporters are important for translocation of lignin precursors across the actual plant membrane. Monolignol transport is assumed to be mediated by ABC transporters and some studies suggested candidate genes^17,27–30^. Previously, transport of coniferyl alcohol and sinapyl alcohol by an ABC-like transporter was demonstrated by using microsomal vesicles obtained from *A*. *thaliana* leaves^15^. An ABC transporter in *A*. *thaliana* was identified as a *p*-coumaryl alcohol transporter and confirmed by heterologous expression in yeast^31^; however, we could not detect transport activity for *p*-coumaryl alcohol in membrane vesicles from differentiating xylem of hybrid poplar and Japanese cypress. The present results strongly suggest that the major route for H lignin monomer transport in differentiating xylem is the proton gradient-dependent transport of *p*-glucocoumaryl alcohol in both gymnosperms and angiosperms. Feeding radio-labelled *p*-glucocoumaryl alcohol enabled incorporation of radioactive labels into the cell wall of differentiating xylem^6–9^, indicating that *p*-glucocoumaryl alcohol is utilised for lignification. The transport activity observed in the present study may play a major role in lignification of xylem tissue.

Experiments using a variety of inhibitors revealed that V-ATPase and the proton gradient are involved in *p*-glucocoumaryl alcohol transport in both hybrid poplar and Japanese cypress (Fig. 4). No inhibition was induced by vanadate, a common inhibitor of ABC transporters. Furthermore, fractionated membrane analysis indicated that the origin of the membranes involved in *p*-glucocoumaryl alcohol transport was not the plasma membrane but the tonoplast and endomembrane (Fig. 5). These results indicate that *p*-glucocoumaryl alcohol is transported by a proton antiporter consuming a proton gradient generated by V-ATPase, which may be a common feature in differentiating xylem of both gymnosperms and angiosperms. It is known that V-ATPase localises to the endomembrane system broadly including vacuoles, ER, Golgi apparatus, *trans*-Golgi network, and other small vesicles^32,33^. These membrane elements are fractionated in light membrane fractions (Fig. 5B), which is consistent with the transport activity of *p*-glucocoumaryl alcohol, suggesting that the transporter responsible for *p*-glucocoumaryl alcohol translocation is enriched especially in the lightest membrane fraction (0/20%) (Fig. 5A). No marker enzyme tested in the current study showed enrichment especially in the lightest membrane fraction (Figs 5, S3). The lightest membrane fraction may contain not only tonoplasts but also endomembranes associated with secretory systems. *p*-Glucocoumaryl alcohol might be loaded into the secretory vesicles, and then delivered to the developing cell wall by exocytosis, as previously proposed for coniferin transport in woody plants^16,18^.

This transport mechanism of monolignol glucosides in differentiating xylem differs from the transport of monolignol glucosides by ABC transporters in *A*. *thaliana* leaves^15^. The ABC transporter family is composed of more than 120 members, some of which may show broad specificity for transport substrates. ABC transporters are presumed to play important roles in the detoxification of xenobiotic compounds and toxic endogenous metabolites^34^. It is reasonable that transport mechanisms of lignin precursors are different between leaves containing mostly mesophyll cells, where lignin precursors are likely to be transported for detoxification, and the differentiating xylem, where lignin precursors should be transported for lignification. Thus, plants may show several transport mechanisms for lignin precursors depending on the species, tissues and/or development stages. The results of the present study suggested that a proton antiporter in tonoplasts and endomembranes are involved in the transport of monolignol glucoside in the differentiating xylem of woody plants.

To examine the specificity of the *p*-glucocoumaryl alcohol transporter, *cis*-inhibition was tested using several compounds (Table 1). Although syringin and sucrose did not cause inhibition of *p*-glucocoumaryl alcohol transport, *p*-coumaryl alcohol and coniferin showed significant inhibition, indicating that the *p*-glucocoumaryl alcohol transporter recognises the structure of phenyl groups. Inhibition of *p*-glucocoumaryl alcohol transport by high concentrations of coniferin is likely to be the result of competitive recognition of the transporter for these similar substrates. Coniferin was indicated to be a mixed-type inhibitor of *p*-glucocoumaryl alcohol transport (Fig. S4), suggesting that coniferin binds to the recognition and other sites on the transporter. Both *p*-glucocoumaryl alcohol and coniferin may be transported by an identical transporter that is conserved in coniferous and broad-leaved trees. Additional studies are required to identify the transporters of lignin precursors and to elucidate the transport mechanism of lignin precursors in differentiating xylem.

## Materials and Methods

### Chemicals

Chemicals used in this study were purchased from Nacalai Tesque (Kyoto, Japan) or Wako Pure Chemicals (Osaka, Japan). Coniferin and syringin were provided by Dr. Noritsugu Terashima of Nagoya University. *p*-Coumaryl alcohol and its glucoside were synthesised as described previously^25,35^.

### Plant materials

An approximately 40-year-old hybrid poplar (*Populus sieboldii* × *P*. *grandidentata*) and an approximately 30-year-old Japanese cypress (*Chamaecyparis obtusa*) were felled in late July and late August, respectively. The differentiating xylem was sampled as described previously^16^; briefly, logs were debarked and the differentiating xylem was scraped off, frozen with liquid nitrogen and stored at −80 °C until required.

### Histochemical analysis

The differentiating xylem of poplar was fixed in 2.5% glutaraldehyde in 0.1 M phosphate buffer (pH 7.2). Transverse sections 40 µm thick were then prepared. For the Mäule reaction, sections were reacted with 1% (w/v) KMnO_4_ solution for 5 min followed by a brief wash with distilled water. The sections were then incubated in 2 M HCl for 5 min followed by a wash with distilled water. Sections were treated with concentrated NH_3_ solution.

### Characterisation of lignin

Serial tangential sections were prepared in accordance with a previously described method^36^ with slight modifications. Briefly, blocks consisting of outer bark tissues, differentiating xylem and a few annual rings were collected from the hybrid poplar and frozen in liquid nitrogen. The frozen specimen blocks were fixed on the freezing stage of a cryotome, and 50-µm-thick serial tangential cryosections were obtained from the bark to the mature xylem. Four serial sections were placed into one vial, which was weighed before and after collecting the sections. The sections were extracted with methanol and acetone followed by drying and weighing. Sections with the lowest dry weight (section 0) from each block were used to sequence the data obtained from each section of the different blocks.

Extracted samples were used to characterise lignin using the microscale thioacidolysis method^37^ with modifications. Briefly, samples were incubated in a glass vial in 0.9 mL of the reaction mixture (dioxane/ethanethiol (9:1, v/v), 92 mM BF_3_ etherate) at 100 °C for 4 h. Sections from two blocks (~2 mg) were used for one reaction. After cooling on ice, 10 *μ*L tetracosane (2 mg mL^−1^ in dichloromethane) was added to the reaction mixture as an internal standard. Then, 400 *μ*L of the reaction mixture was transferred to a 1.5 mL siliconised polypropylene tube. After addition of 200 *μ*L of 1 M NaHCO_3_, the solution was adjusted to pH 3–4 using 3 M HCl. To the solution, 300 *μ*L diethyl ether was added, and the tube was vortexed and centrifuged to obtain the organic layer. The neutralisation and extraction were repeated twice using another 400 *μ*L of the reaction mixture. The collected organic layer was concentrated and analysed by GC-MS as described in a previous study^25^.

### Preparation of microsomal fractions

Microsomal fractions were prepared from the scraped xylem in accordance with a previously described method^16,22^. Briefly, frozen sample of differentiating xylem (~10 g) from each tree specimen was homogenised with a mortar and pestle for 10 min in approximately 20 mL homogenizing buffer [10% (v/v) glycerol, 0.5% (w/v) polyvinylpolypyrrolidone, 5 mM EDTA, 50 mM HEPES–KOH (pH 8.0), 150 mM KCl, 3.3 mM dithiothreitol and 1 mM phenylmethylsulfonyl fluoride]. The homogenate was filtered through Miracloth (Merck, Rahway, NJ, USA) and centrifuged at 3,600 × *g* for 10 min. The supernatant was centrifuged at 136,000 × *g* (80Ti, Beckman, Palo Alto, CA, USA) for 30 min. The microsomal pellet was resuspended with resuspension buffer [10% (v/v) glycerol, 1 mM EDTA and 10 mM HEPES-KOH (pH 7.6)] and centrifuged under the same condition (136,000 × *g*, 30 min). The microsomal pellet was resuspended in resuspension buffer. All procedures described above were performed on ice or at 4 °C. The microsomal fraction was immediately frozen with liquid nitrogen and stored at −80 °C.

### Measurement of transport activity

A transport assay was conducted as reported previously^16,22^. Uptake of lignin precursors by membrane vesicles was measured at 28 °C for 20 min in 100 *μ*L reaction mixture [50 mM HEPES-KOH (pH 7.5), 5 mM Mg/ATP, 50 *μ*M lignin precursors and membrane vesicles (~15 *μ*g protein)], unless otherwise stated. Eighty microliters of the reaction mixture were filtered using a Sephadex G-50 spin column and centrifuged at 2,000 rpm for 2 min. An equal volume of methanol was added to the filtrate for HPLC analysis. Ten microliters of each sample were injected onto a reversed-phase Inertsil^®^ ODS-4 column (150 × 4.6 mm i.d., 5 µm; GL Sciences, Tokyo, Japan) maintained at 30 °C. The flow rate was 1.0 mL min^−1^ throughout the entire analysis with 0.1% (v/v) acetic acid and 15%, 25%, or 30% (v/v) methanol for determination of *p*-glucocoumaryl alcohol, *p*-coumaryl alcohol, or *p*-coumaric acid, respectively. The peaks of lignin precursors were detected by measuring the absorbance at 260 nm. Ammonium chloride and vanadate were dissolved in water, whereas bafilomycin A1 and gramicidin D were dissolved in DMSO, and nigericin was dissolved in methanol. In the control, DMSO or methanol was added to the reaction mixture to a final concentration of 1%. Data are given as technical replicates.

### Fractionation of membrane vesicles

For fractionation of the microsomal membrane vesicles, the microsomal fraction obtained above was layered onto a discontinuous gradient containing 20–50% (w/v) sucrose in centrifugation buffer [1 mM EDTA, 10 mM HEPES–KOH (pH 7.6)] in a 5 mL centrifugation tube. The gradient was then centrifuged at 237,000 × *g* for 1 h (SW55Ti, Beckman), and each fraction collected from the interface was resuspended in resuspension buffer followed by centrifugation at 136,000 × *g* (80Ti, Beckman) for 30 min. The microsomal pellet of each fraction was resuspended in resuspension buffer.

### Protein gel blotting

For immunoblotting, hybrid poplar membranes were incubated with 0.1% (w/v) *n*-dodecyl-*β*-d-maltoside for 10 min followed by denaturation in buffer [5 M urea, 2% (w/v) SDS, 1.6% (v/v) 2-mercaptoethanol, 100 mM Tris-HCl (pH 8.0)] for 10 min at room temperature. Denatured membrane proteins (8 µg per lane) were subjected to SDS-PAGE in a 7% polyacrylamide gel and then transferred to a polyvinylidene difluoride membrane. The transferred membrane was blocked and incubated with primary antibodies overnight at 4 °C. Primary antibodies used were raised against PIP, V-ATPase and ER luminal BiP (Cosmo Bio, Tokyo, Japan). After washing, the membrane was incubated with secondary alkaline phosphatase-conjugated anti-rabbit IgG antibody (MP Biomedicals, Solon, OH, USA). The band was visualised by reaction with nitro-blue tetrazolium and 5-bromo-4-chloro-3′-indolyl phosphate.

## Supplementary information

Supplementary Dataset 1

## Data Availability

All data generated or analysed during this study are included in this published article and Supplementary Information files.
