# Supplementary material for: Proton Gradient-Dependent Transport of p-Glucocoumaryl Alcohol in Differentiating Xylem of Woody Plants
Source: Sci Rep. 2019 Jun 20;9:8900. doi: 10.1038/s41598-019-45394-7 (PMC6586893; doi:10.1038/s41598-019-45394-7)
Supplement: Supplementary file 1 — Supplementary Dataset 1 [file 41598_2019_45394_MOESM1_ESM.pdf]

Proton Gradient-Dependent Transport of *p*-Glucocoumaryl Alcohol in Differentiating Xylem of Woody Plants

Taku Tsuyama[1*], Yasuyuki Matsushita[2], Kazuhiko Fukushima[2], Keiji Takabe[3], Kazufumi Yazaki[4], Ichiro Kamei[1]

[1]Faculty of Agriculture, University of Miyazaki, Miyazaki 889-2192, Japan

[2]Graduate School of Bioagricultural Sciences, Nagoya University, Nagoya 464-8601, Japan

[3]Graduate School of Agriculture, Kyoto University, Kyoto 606-8502, Japan

[4]Research Institute for Sustainable Humanosphere, Kyoto University, Uji 611-0011, Japan

***Corresponding Author:**

T. Tsuyama

Faculty of Agriculture, University of Miyazaki, 1-1 Gakuen-kibanadai-nishi, Miyazaki 889-2192, Japan

Tel.: 81-985-58-7182 Fax: 81-985-58-7182 E-mail: tsuyama@cc.miyazaki-u.ac.jp

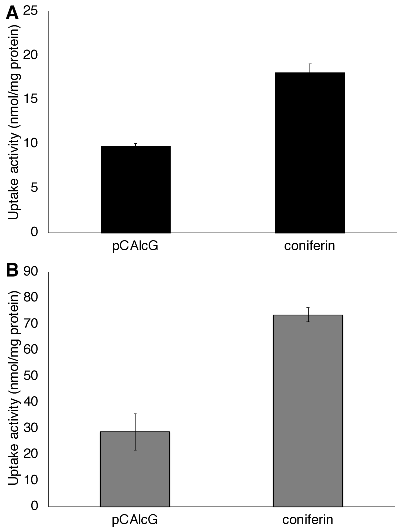

Figure S1. Uptake of lignin precursors into membrane vesicles obtained from differentiating xylem of hybrid poplar (*Populus sieboldii* × *P. grandidentata*) (A), Japanese cypress (*Chamaecyparis obtusa*) (B), respectively. Membrane vesicles were incubated with 50 μM of each compound in the presence of 5 mM ATP for 20 min. pCAlcG; *p*-glucocoumaryl alcohol. Data are means ± SD of three replicates.

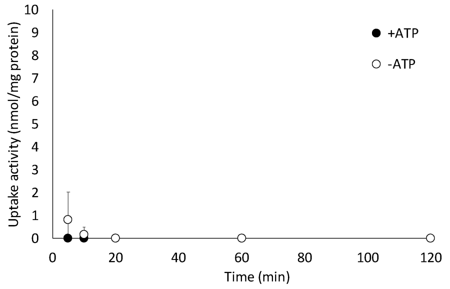

Figure S2. A time course of *p*-coumaryl alcohol uptake in hybrid poplar membrane vesicles. Membrane vesicles were incubated with 50 μM of *p*-coumaryl alcohol in the presence (●) or absence (○) of 5 mM Mg/ATP. Data are means ± SD of three replicates.

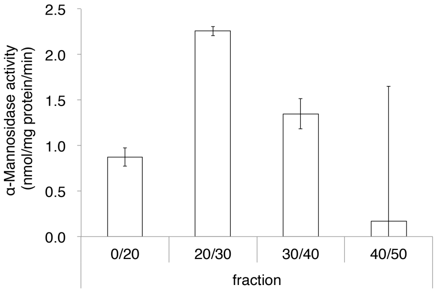

Figure S3. Alfa-mannosidase activity in sucrose gradient-fractions of hybrid poplar was used as a marker of central vacuole[24]. Membrane fractions were incubated with 5 mM *p*-nitrophenyl-α-D-mannopyranoside in 100 mM sodium citrate buffer (pH 4.6) for 1 h, followed by measurements of absorbance at 400 nm for determination of liberated *p*-nitrophenol. Data are means ± SD of three replicates.

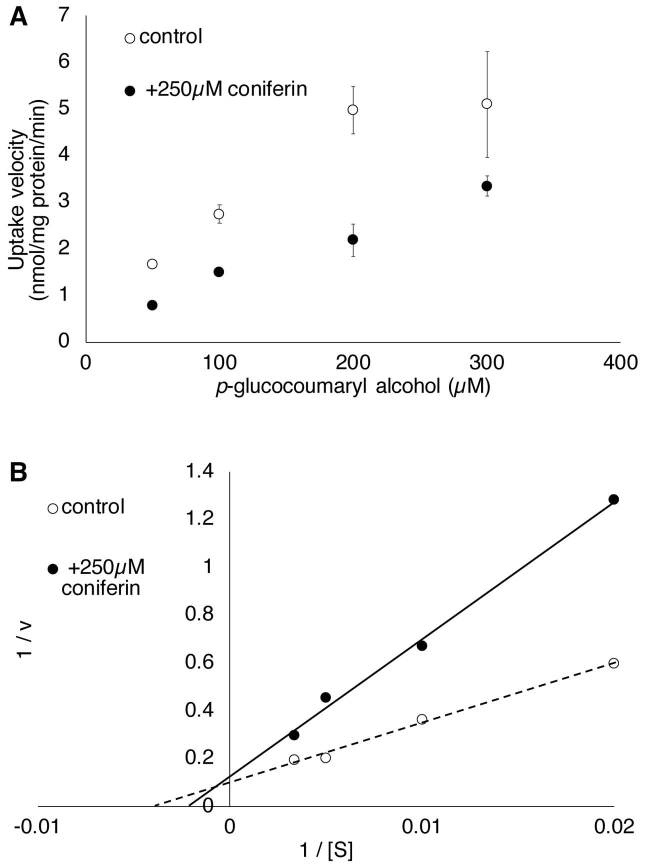

Figure S4. Mixed-inhibition of *p*-glucocoumaryl alcohol transport by coniferin. (A), Membrane fractions of hybrid poplar were incubated for 5 min in the presence of 5 mM Mg/ATP and each concentration of *p*-glucocoumaryl alcohol with (●) or without (○) 250 μM coniferin. Data are means of three replicates. (B), Lineweaver-Burk plots of results in (A). Calculated apparent K_m values were 256 μM (control) or 451 μM (+250 μM coniferin) and calculated V_{max} values were 10.14 nmol・mg protein^{-1}・min^{-1} (control) or 7.86 nmol・mg protein^{-1}・min^{-1} (+250 μM coniferin).

Table S1. K_m values of the *p*-glucocoumaryl alcohol uptake for comparison with respect to secondary transporters

Compounds	plant species	K_m
p-glucocoumaryl alcohol	hybrid poplar	160-260 μM*
coniferin	hybrid poplar	60-80 μM[16]
coniferin	Japanese cypress	24-26 μM[16]
isovitexin	barley	82 μM[19]
saponin	barley, *Arabidopsis*	50–100 μM[20]
salicylic acid 2-*O*-D-glucoside	tobacco	11 μM[21]
berberine	*Coptis japonica*	43.7 μM[22]
salicylic acid glucose ester	*Arabidopsis thaliana*	38 μM[23]

*, present study.
